# Early warning climate indices for malaria and meningitis in tropical ecological zones

**DOI:** 10.1038/s41598-020-71094-8

**Published:** 2020-08-31

**Authors:** Ayansina Ayanlade, Isioma J. Nwayor, Consolato Sergi, Oluwatoyin S. Ayanlade, Paola Di Carlo, Olajumoke D. Jeje, Margaret O. Jegede

**Affiliations:** 1 Department of Geography, Obafemi Awolowo University, Ile-Ife, Nigeria; 2 University of Alberta, Edmonton, Canada; 3 African Institute for Science Policy and Innovation, Obafemi Awolowo University, Ile-Ife, Nigeria; 4 PROMISE Department, University of Palermo, Palermo, Italy

**Keywords:** Climate sciences, Diseases

## Abstract

This study aims at assessing the impacts of climate indices on the spatiotemporal distribution of malaria and meningitis in Nigeria. The primary focus of the research is to develop an Early Warning System (EWS) for assessing climate variability implications on malaria and meningitis spread in the study area. Both climate and health data were used in the study to determine the relationship between climate variability and the occurrence of malaria and meningitis. The assessment was based on variations in different ecological zones in Nigeria. Two specific sample locations were randomly selected in each ecological zone for the analysis. The climatic data used in this study are dekadal precipitation, minimum and maximum temperature between 2000 and 2018, monthly aerosol optical depth between 2000 and 2018. The results show that temperature is relatively high throughout the year because the country is located in a tropical region. The significant findings of this study are that rainfall has much influence on the occurrence of malaria, while temperature and aerosol have more impact on meningitis. We found the degree of relationship between precipitation and malaria, there is a correlation coefficient R^2^ ≥ 70.0 in Rainforest, Freshwater, and Mangrove ecological zones. The relationship between temperature and meningitis is accompanied by R^2^ ≥ 72.0 in both Sahel and Sudan, while aerosol and meningitis harbour R^2^ = 77.33 in the Sahel. The assessment of this initial data seems to support the finding that the occurrences of meningitis are higher in the northern region, especially the Sahel and Sudan. In contrast, malaria occurrence is higher in the southern part of the study area. In all, the multiple linear regression results revealed that rainfall was directly associated with malaria with β = 0.64, p = 0.001 but aerosol was directly associated with meningitis with β = 0.59, p < 0.001. The study concludes that variability in climatic elements such as low precipitation, high temperature, and aerosol may be the major drivers of meningitis occurrence.

## Introduction

Climatic variability and seasonality play a significant role in the spatiotemporal distribution of diseases. It has been reported that about 700,000 to 2.7 million people die of malaria each year, the majority of them, nearly 75% of those, are African children^1^. Several studies have shown that the occurrence and spatial distribution of malaria are sensitive to the seasonality of climatic factors in Iran, most African countries, and other parts of the world with significant perinatal morbidity and mortality^2–6^. Disease vectors depend on suitable habitats to breed, which in turn depends heavily on climatic conditions and for understanding the nature of some illness^7^. The malaria and meningitis (MM) transmission is highly seasonal due to climatic conditions; these occurrences are much more frequent in recent times due to climate change^8–14^. Climate and health are indistinctly interconnected, and this is the same for infectious diseases^15,16^. Climate change is likely to increase malaria^13,17,18^ and meningitis^19–21^ incidence as the future environment might become more suitable for malaria transmission in many tropical highlands. There is a corresponding 0.90% increase in the number of malaria cases to each 1 °C temperature increase^22^. Molecular epidemiology and whole-genome sequencing analysis studies of Neisseria meningitis isolate in Nigeria have shown that not only the virulence of a strain influences the trend of invasive forms of the meningococcal disease but also climate changes are involved^23–25^.

Some studies in Sub-Saharan Africa have reported an increase in the number of malaria cases with changes in climate^26–28^. For example, a study by Grover-Kopec et al.^26^ further stated that the major public health problem in many Sub-Saharan African countries is the periodic epidemics of malaria, which is common in the area with poorly developed immunity to malaria. Malaria is a significant public health problem in many Sub-Saharan African countries, with nearly 200 million clinical episodes and almost one million deaths occurring annually^26,29^. Meningitis occurs mostly during the warmer months between February and April, with its significant effect on a large number of people in cycles of 8 to 12 years^30^. Besides, studies have reported that hot deserts storm increases the risk of meningitis in Africa, especially in the Sahel region of West Africa which is immediately after the Sahara desert^31,32^. Many of the recent studies have reported the need for climate information, as an interdisciplinary approach, for better public health action^33–35^. The health decision-makers gradually search for a better understanding of the climate as it relates to public health^36–39^.

Consequently, understanding variability and changes in climatic indices are essential for a proper evaluation of their impacts on human health. The relationship between climatic indices and the epidemics remain inconclusively investigated^40–42^. The change in the MM epidemics over different ecology ecological is a significant gap. For this reason, this study aims at examining the relationship between variability in climatic indices: (precipitation, temperature, and aerosol) and the occurrences of malaria and meningitis across tropical ecological zones in Nigeria. The study is based upon the assumption that climate does not only define the seasonal and spatial distribution of MM, but it also influences the inter-annual variability of MM epidemics and their long term trends^15,43–45^. As a consequence, climate variability is now an issue of concern for health policy in many countries because the total number of people at risk, the age structures of the population and the density of the settlement are essential variables in determining the socio-economic development at any country^46–48^.

## Materials and methods

### The study area

**Figure 1:**
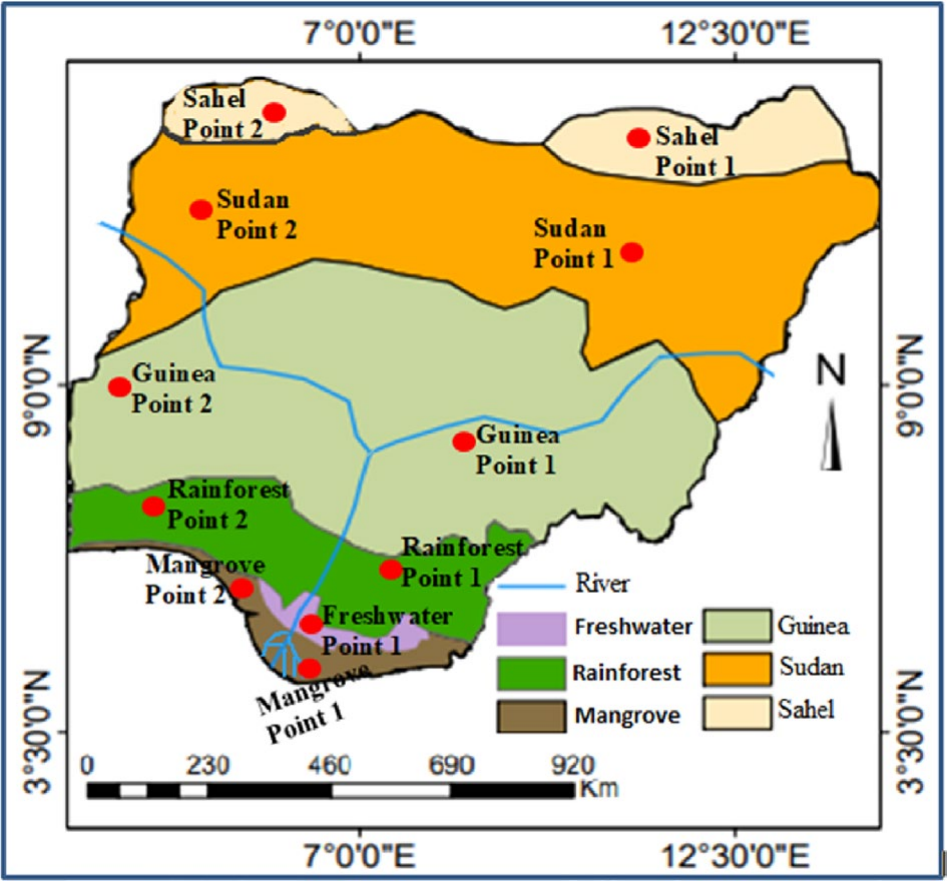
Map of the study area, showing distinct ecological zones with specific locations of the data collection. Two locations were purposefully sampled per ecological zone as in the red points. The figure was generated using ArcGIS 10.5 software (https://www.esri.com/).

This study involves different ecological zones in Nigeria (Fig. 1). Nigeria is a tropical country located approximately between 4°N and 14°N latitudes and 3°E and 14°E longitudes in West Africa, covering a total area of about 923,768 sq. Km. Cameroon, Niger Republic, and Republic of Benin border the country in the East, North, and West, respectively (Fig. 1), while Nigeria is surrounded by the Gulf of Guinea (Atlantic Ocean) in the south^49^. The study area experiences abundant rainfall accompanied by convectional storms, having annual precipitation of about 15,000 mm and a mean minimum temperature between 18 °C and 36 °C. In the north, the mean temperature is higher compared to the west and south, where the temperature is curbed by the influence of rainfall, which reduces northward over the country. The seasonality of climate is influenced by the variability of the Inter-Tropical Convergence Zones (ITCZ) across the country. The ITCZ is formed by the actions of the north-easterly and south-westerly air masses^50^. The study sites are the six typical ecological zones in this country, transiting in the north–south direction from the fringe of Sahel savannah to the Atlantic coast. These are the Sahel Savannah, Sudan Savannah, Guinea Savannah, Rainforest, Freshwater, and the Mangrove swamp. Rainfall per annum varies in these ecological zones, from as high as 3000 mm in the Mangrove swamp to about 500 mm in the Sahel savannah.

### Data acquisition

Satellite climatology data was used in this study. The climatic data used in this study were retrieved satellite observation, including precipitation, minimum and maximum temperature from January 2000 to December 2018, and aerosol optical depth from January 2000 to December 2018. These data were used to develop climate indices for the early warning system for MM in Nigeria. Satellite climatology dataset acquired from the National Oceanic and Atmospheric Administration (NOAA) while the International Research Institute for Climate and Society (IRI), New York provides the initial training on satellite data acquisition and climate data analysis. Both kinds of satellites are necessary for providing a complete global weather monitoring system. A different set of data used includes the Moderate Radiometer Imaging Spectroradiometer (MODIS-Terra and Aqua) and dekadal (10-day) precipitation estimates from the Climate Prediction Center. The data were made freely available on the IRI web. The data for each climate variable were downloaded from IRI web. https://iridl.ldeo.columbia.edu/maproom/Health/Regional/Africa/Malaria/System.html. MODIS, an imaging instrument that flies on two satellites in NASA’s (National Aeronautics and Space Administration) Earth Observing System. Some of the characteristics of MODIS are; Spatial resolution: 250 m (bands 1–2), 500 m (bands 3–7), 1000 m (bands 8–36), temporal resolution: 1 to 2 days in 36 discrete spectral bands. NOAA has the following abilities that are essential for early warning system: Improved short-term warning and forecasting services, forecasting climate trends and changes. The major benefit of these datasets is that satellite observation data can be used for global analysis beyond the study area; it also provides a good spatial and temporal resolution across Nigeria.

For practical evaluation, both climatic data and epidemic (malaria and meningitis) were analysed. Data from MODIS databases were assessed. The analysis of climate indices was carried out using dekadal precipitation and precipitation estimate percentages. The MM data covers the period 2000 to 2018 such as climate data. The MM cases were summed over two age groups (under and over 5 years). Some of the datasets were updated with the dataset from the Nigerian National Bureau of Statistics (NBS), with monthly MM data and regional informations. The NBS generates all set of statistics for the country on a continuous and sustainable basis. Each ecological zone is a vast area covering more than two states. Thus two locations were purposefully sampled per environmental zone. Since the data is used for research only and involving no human participants, permission to use the data was granted by NBS.

### Statistical analysis

In this study, other climatic variables such as relative humidity and wind speed were applied. The principal climate variables usually considered are concerning rainfall, temperature, aerosol, wind speed, and humidity^52^. The specific rainfall indices which were used in this present study are primarily based on the work by Nigussie and Altunkaynak^53^ and Muluneh, et al.^54^. 7 days were adopted, which have highest adequate precipitation during a consecutive 14-days period, and highest adequate precipitation during the following 30-days period. In the present study, we categorized a “rain-day” as a period of twenty-four hours with total rainfall equal to or greater than the minimum measurable amount and a “dry-day” as a period of twenty-four hours with no rainfall. The critical temperature indices used in this study were based on Rahimi and Hejabi^55^. The temperature indices used included daily mean temperature, mean daily maximum temperature, mean daily minimum temperature, maximum consecutive days with daily maximum temperature above 15 °C in days, and warm/hot nights frequency: percentage of days when daily minimum temperature (TN) 90th percentile. Calculation of percentage occurrence of climate conditions suitable for the transmission of MM was initiated based on climate indices. Descriptive statistical techniques were adopted; maps and charts were developed to show the estimate and trend of climatic indices, which includes precipitation, aerosols optical depth, and maximum and minimum temperature. The pattern of these climatic indices was compared with MM dataset to show the relationship between the diseases and climatic indices. An Early Warning System (EWS) model was developed to project the possibility of MM occurring at specific periods of climatic extremes. Numerous climate indices had been projected for climate change, and it impacts on health^51^. The annual cases of MM from health facilities in sampled local government areas of Nigeria were used in a novel stratification process. The aerosols-optical depth suspended in the atmosphere influenced by the action of wind and precipitation/ITCZ in Nigeria was estimated. Studies have provided threshold that specific indices are estimated on the outbreak and prevalence of MM at a particular location. Concerning malaria, limits are assumed as average precipitation more significant than 80 mm, temperature ranging from 18 °C and 32 °C, and relative humidity (RH > 60%)^56^. Meningitis, on the other hand, seems to prevail with high temperature, high aerosol, and a threshold of dryness reaching 40%^20,57^. In the present study, the probability of the occurrence of MM concerning climate variability was further estimated as the “early warning system”. Probability analysis was based on observations of meningitis events, the Meningitis Forecasting for Africa Project (MFAP), as presented in IRI web https://iridl.ldeo.columbia.edu/SOURCES/.LSTM/.MFAP/.molesworth_etal_2003/searches.html?bbox=bb%3A0.250%3A2.361%3A14.849%3A14.291%3Abb . MFAP is an investigate model meningitis events or probability that leads forecast of meningitis occurrence. Details information about MFAP were reported by Molesworth et al.^58^. MFAP demonstrates the probability of meningitis occurrence and changes as climatic indicators changes over different ecological zones. The model present the probability rates of meningitis epidemic experience for each ecological zones.

To assess the relationship between climatic indices and the epidemic of infectious diseases understudy, a correlation analysis was performed. Spearman correlation coefficient (R^2^) was used to estimate the relationship between precipitation, and aerosol with MM in the six major points from each ecological zones. Multivariable regression was carried out to investigate the relationship between the MM and each climatic variable. The choice of the multivariate regression analysis was started by examining the nature of the variables used in the study, using Quantile–Quantile (Q–Q) plots. It is clear from the Q–Q plots that the outcome is continuous and follows a normal distribution with a positive slope. Since the Q–Q plots indicated that the outcome variable followed a normal distribution, a multiple linear regression model was therefore used. The multiple linear regression models were based on Eq. (1):

1 $${\text{y}}_{{{\text{Malaria}}/{\text{Meningitis}}}} =\upbeta _{0} +\upbeta _{{1}} {\text{x}}_{{1}} +\upbeta _{{2}} {\text{x}}_{{2}} +\upbeta _{{3}} {\text{x}}_{{3}}$$

where y is the malaria/meningitis, x is the climatic variables (x_1_ = rainfall, x_2_ = temperature , x_3_ = aerosol), and β is the coefficient for each climatic variables respectively. During multivariate regression analysis, the interrelationships among all climatic variables were also taken into account to assess the significant impacts of each climatic variable on MM. The R Squared was used as the proportion of variability in the MM which can be explained by the climatic variables in the model.

## Results and discussion

**Figure 2:**
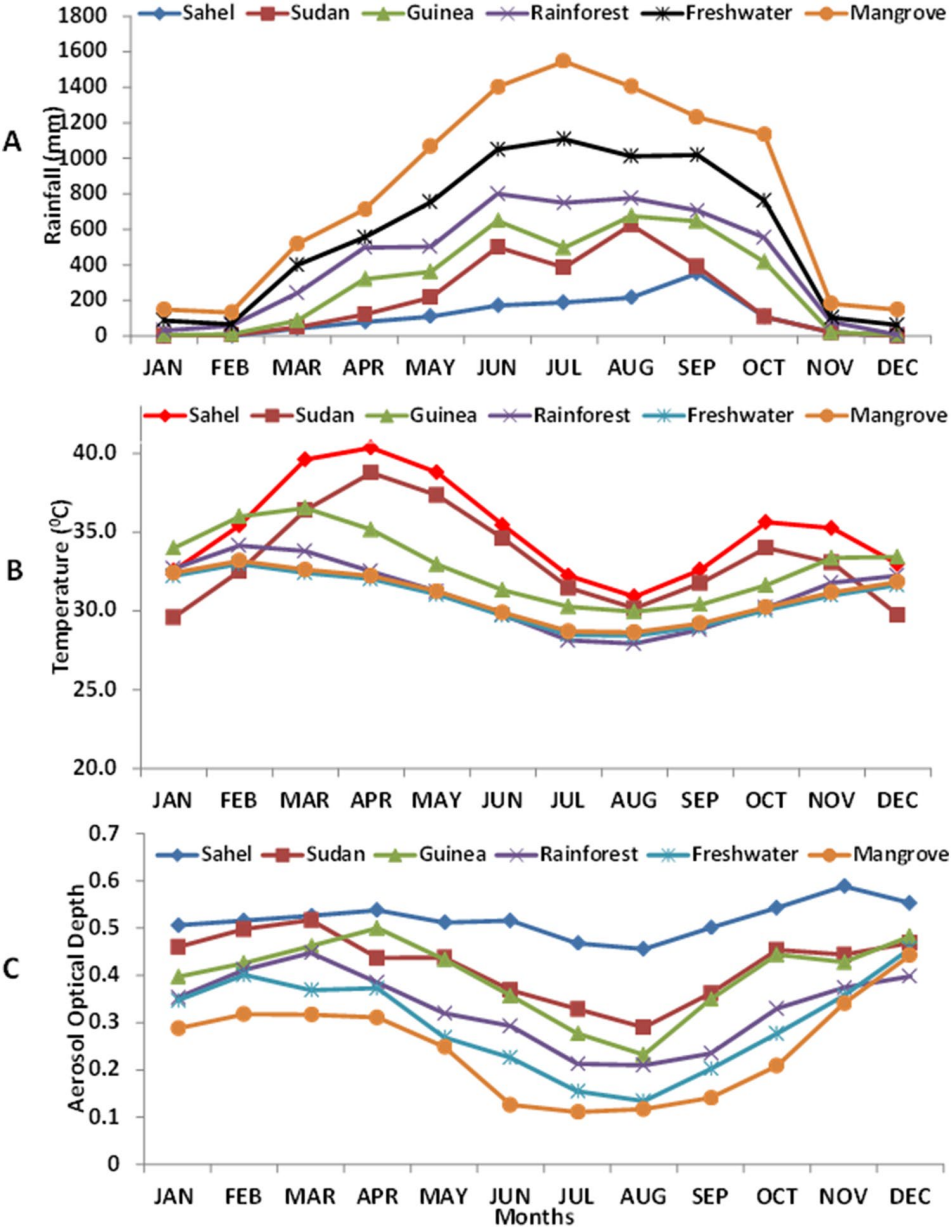
Mean monthly variation in rainfall (**A**), temperature (**B**) and aerosol (**C**) over different ecological zones.

The result from this research shows three significant findings: (1) occurrence of Climatic Indices vary across ecological zones; (2) the Climatic Index with the most considerable influence on the appearance and spread of malaria is precipitation; and (3) with regard to meningitis, temperature and aerosol are the most significant climate indices. Although during initial analysis, we applied other climatic variables such as relative humidity, wind speed, we find out their relationship is very weak, but the initial test revealed rainfall, temperature, and aerosol were significantly associated with MM (p < 0.01). Variations in climatic indices in the ecological zone are noticeable from dry to wet seasons (Table 1). In general, the mean monthly temperature ranges from 29 °C to 42 °C in all ecological zones, but much more high in the Sahel and Sudan (Fig. 2). The temperature is at its highest (≥ 30 °C) during the dry seasons (Table 1), which are the months from January to March in all the ecological zones (Fig. 2). Most of the months of the year, in both Sahel and Sudan, are hot and dry with maximal temperatures rising as high as 40 °C (Fig. 2B) especially during the month of the dry season (Table 1). During the peak of dry season, especially the months of February and March, the mean monthly temperature ranges between 36 °C and 42 °C in both Sahel and Sudan. The average low temperatures are observed during the months of June, July and August with temperature ranges from 25 to 31 °C (Fig. 2B) in both Sahel and Sudan, however, lower mean monthly temperatures are observed in other ecological zones, about 3 to 5 °C lower (Table 1).

**Table 1:** Variation in climatic indices in the study area for different seasons.

Ecological zones	Maximum temperature (°C) 2002–2018		Minimum temperature (°C) 2002–2018		Average rainfall (mm) 2002–2018		Average aerosol 2000–2018	
	Dry	Wet	Dry	Wet	Dry	Wet	Dry	Wet
Sahel	43.68	31.87	29.36	21.25	54.18	676	0.54	0.49
Sudan	40.13	31.25	29.32	29.71	61.02	749	0.51	0.42
Guinea	32.57	29.43	28.85	27.08	73.37	896	0.40	0.20
Rainforest	31.87	28.63	28.72	27.57	110.65	932	0.26	0.18
Freshwater	30.47	26.32	27.92	26.15	198.58	1,343	0.21	0.15
Mangrove	30.68	25.57	27.59	26.49	219.57	2,708	0.21	0.14

In all ecological zones, precipitation varies from season to season, but it is more pronounced in the Sahel zone with the lowest rainfall (Table 1 and Fig. 2). However, the precipitation appears much more in Rainforest, Freshwater, and Mangrove zones with the peaks in June, July, and August (Fig. 2A). The Sahel zone is a dry land in the Northern part of the study area, with a short rainy season months (Fig. 2A) compared to the Southern region (Rainforest, Freshwater and Mangrove zones), where rainfall is abundant (> 200 mean monthly value) due to the influence of the Atlantic Ocean bordering Nigeria at the south that increases the moisture of the air and causes rain to fall. In both Sahel and Sudan ecological zones, the mean monthly rainfall is very low compared to other zones, with annual rainfall ranges between 100 and 300 mm, and mostly rains during three to six months (Fig. 2A), while the other months may be very dry < 50 mm and some time nor rainfall at all. Generally, the mean monthly rainfall are higher Rainforest, Freshwater and Mangrove zone compared Sahel and Sudan (Table 1 and Fig. 2A ).

The mean monthly amount of aerosol optical depth (AOD) varies from one ecological zone to the others (Fig. 2C). Monthly distribution of aerosol in Sahel savannah is at peak in February and extends to April, 0.40 ≤ AOD ≥ 0.5 (Fig. 2C). Likewise, Sudan Savannah shows a similar trend with Sahel Savannah 0.40 ≤ AOD ≥ 0.5. The disparity with Guinea Savannah and the Sudan Savannah begins (wet season) in April, there is a slight increase in AOD, while May to December experience a more considerable variation than Sahel and Sudan savannah (Table 1 and Fig. 2). In the Rainforest zone, the highest AOD is in April followed by February, while the lowest amount is in the wet season (Table 1), during the month of June to August with 0.1 ≥ AOD ≥ 0.3 (Fig. 2C). The Freshwater ecological zones share a similar occurrence of AOD with the Rainforest ecological zone. The monthly and seasonal distribution of atmospheric aerosols varied over the different ecological zones^59^. It is obvious that the distributions of atmospheric aerosols reflect the climate seasonality. Largely, the observed variations in climatic indices reveal high rainfall during the wet season, with relatively low temperature and low aerosol, whereas there appear to be inverse values during the dry season (Table 1).

### Climate indices and their influences on malaria and meningitis (prevalence/incidence) in different ecological zones

**Figure 3:**
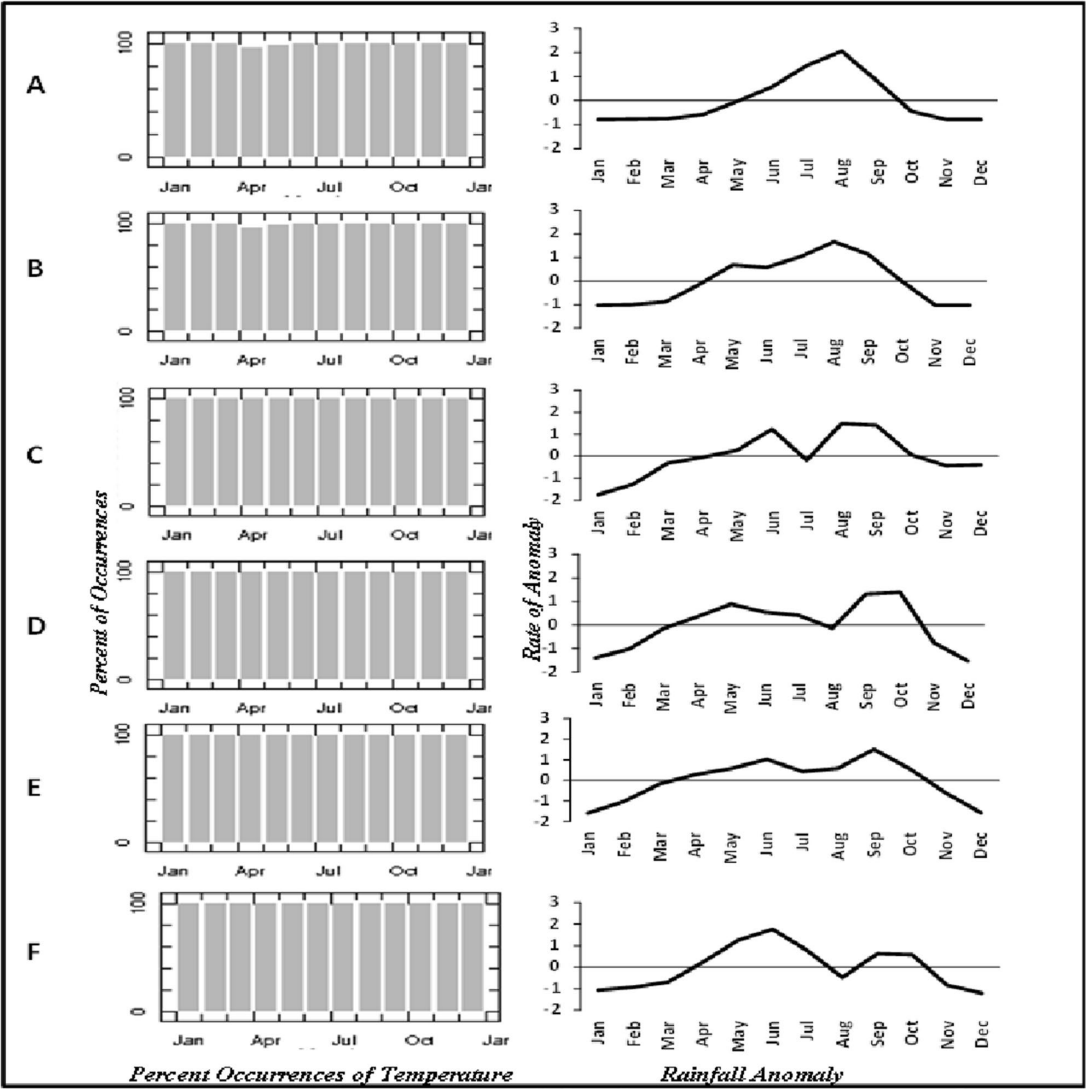
Temperature percent occurrences and rainfall anomaly over different ecological zones: (**A**) Sahel, (**B**) Sudan, (**C**) Guinea, (**D**) Rainforest, (**E**) Freshwater, and (F) Mangrove.

**Figure 4:**
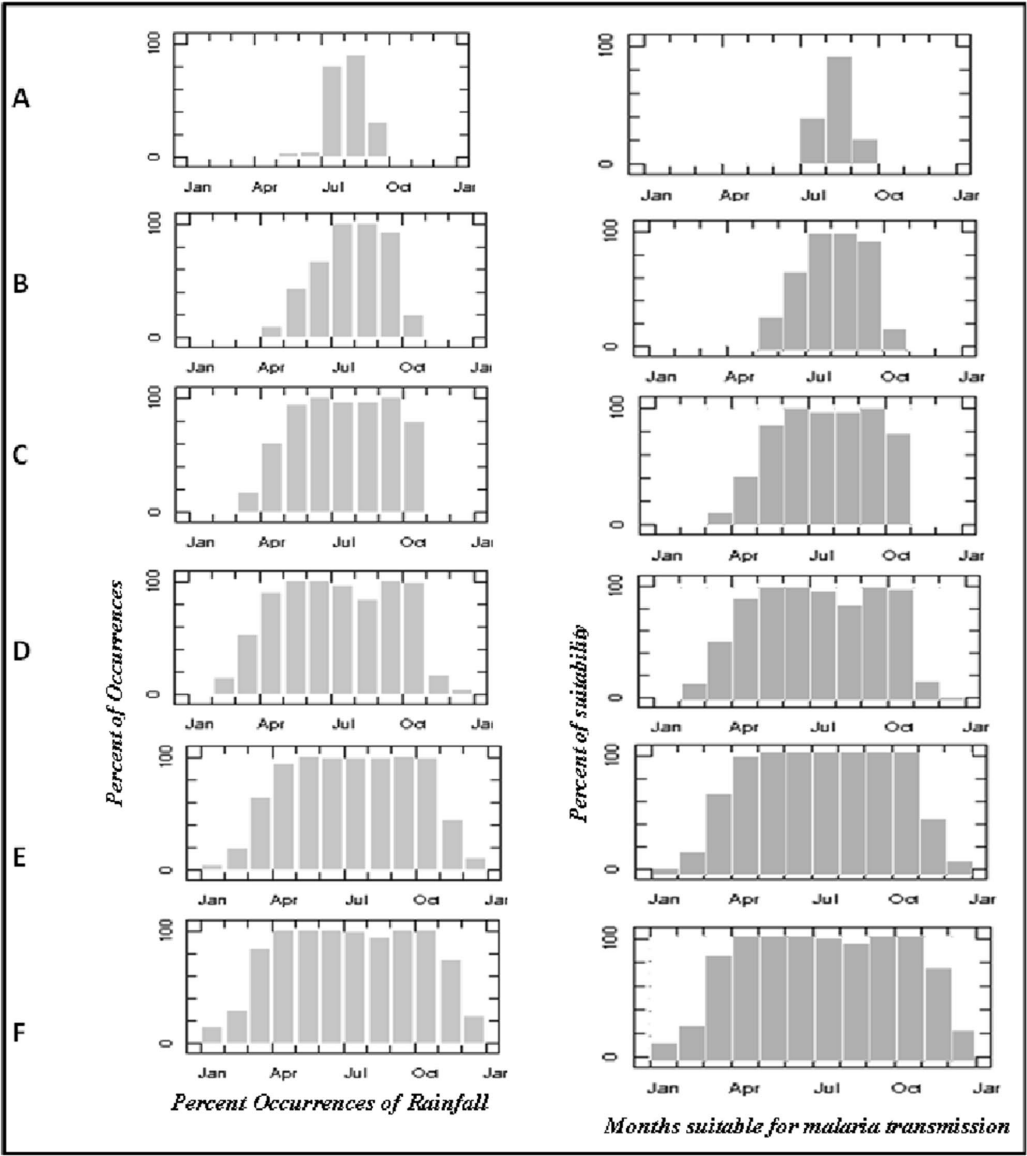
Rainfall percent occurrences and months suitable for malaria transmission over different ecological zones: (**A**) Sahel, (**B**) Sudan, (**C**) Guinea, (**D**) Rainforest, (**E**) Freshwater, and (**F**) Mangrove.

The results of this study reveal that the distribution patterns of temperature occurrences across the ecological zones in Nigeria are distinct (Fig. 3). In Sahel savanna, the temperature decreases from April to June, while the other months display a quite variable temperature amount (Fig. 3A). Sudan savanna indicates a slight dissimilarity in temperature when compared to Sahel savanna and the temperature is relatively high and stable throughout the year with a slight decrease in April (Fig. 3B). The temperature distribution appears the same across the other ecological zones: Guinea savanna, Rainforest, Freshwater, and Mangrove, with a 100% occurrence throughout the year (Fig. 3C–F). The reason for this scenario is apparent. The ecological zones are within the tropical region of the world with little variation in temperature over the year. On the other hand, the precipitation appears more variable across ecological zones with a few anomalies (Figs. 3, 4). Mangrove and Freshwater ecological zones represent the location with much precipitation with rainfall periods spread from May to October. Despite the high precipitation in these regions, November to March exhibit the lowest amount of rainfall (Fig. 3E,F). In the Sahel Savannah precipitation occurs only from May to September, while the other months experience little or no rain (Fig. 4A). Though the Sudan falls directly below the Sahel savannah, precipitation occurrence is relatively higher than in the Sahel savannah, and precipitation occurs from May to October (Fig. 4B). The Guinea savanna experiences a significant amount of variation in the distribution of climate indices, and the ITCZ falls within this region before it is pushed southwards by the north-easterly wind. Precipitation occurrence in the Guinea savannah starts in March and ends in October with the highest amount occurring in June and September (Fig. 4D). The results (Figs. 3D, 4D) shows the rate of precipitation in Rainforest, rainfall is predominant from February to December, the early and late months of the year which are the dry seasons of Nigeria from November to February are the months with the lowest amount of precipitation. The reasons for this is clear since the rainforest is made up of a large proportion of forest trees in Nigeria, and this increases the amount of precipitation in this ecological zone.

**Figure 5:**
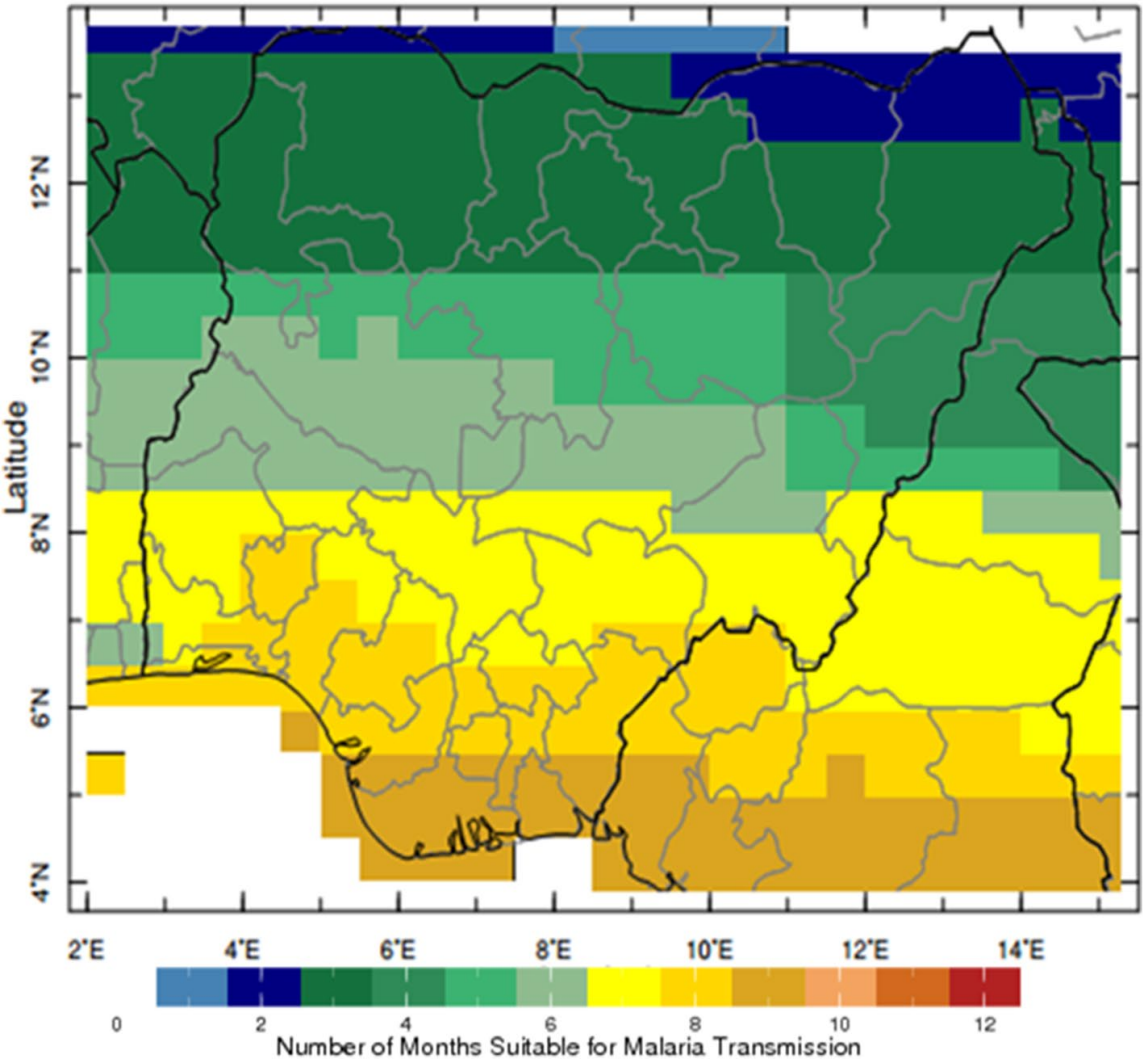
Map of Nigeria showing the number of months suitable for Malaria Transmission. The data were obtained from the IRI database (https://iridl.ldeo.columbia.edu/) while the figure was generated using ArcGIS 10.5 software (https://www.esri.com/).

The percent occurrences of climate conditions suitable for malaria transmission compare the rate of precipitation, and the corresponding amount of rainfall in each ecological zone are presented in Fig. 4. Each ecological zone shows a distinct rate of malaria transmission. Precipitation is higher in the southern region of Nigeria than in the north, and this is due to the movement of moisture-laden air mass generated from the Atlantic Ocean in the south. For the first three ecological zones in the north: Sahel Savannah, Sudan Savannah and Guinea Savannah, the percent occurrence of climates conditions suitable for malaria transmission is relatively lower (Fig. 4A–C) than the southern part of Nigeria: Rainforest, Freshwater and Mangrove (Fig. 4D–F). The results of the climate indicator and suitable for malaria transmission also revealed variations for each location (Fig. 5). The pattern of malaria transmission in Fig. 4 shows disparity throughout the ecological zones, the months suitable for malaria transmission are more in the south than in the north (Fig. 5). In Sahel savannah down to Guinea savannah, the months ideal for the transmission of malaria ranges between 1 to 5 months. The case in southern Nigeria is quite different as the months ideal for malaria transmission range between 5 to 9 months (Fig. 5). The reasons for this variation are obvious in each ecological zones, there is variation in the distribution of climatic indices for each season. Different ecological zones have a varied amount of temperature and precipitation (Table 1). Generally, rainfall is more abundant in the south than in the north. The north regions experience higher temperatures than the south. AOD is more significant in Sahel and Sudan savanna, and it reduces downwards to the south of Nigeria.

### The relationship and impacts of climatic indices on the occurrence of malaria and meningitis

The relationship between climate indicators and circumstances of MM are presented in Table 2. What is evident from the results is that malaria exhibits the strongest correlation with precipitation. The results imply that as precipitation increases, the breeding ground for adult mosquitos’ expand^5^. Meningitis is most affected by dust (aerosol). The influence of temperature on meningitis is most prominent in the Sahel with a Spearman correlation coefficient R^2^ = 78.45. The influence of temperature on malaria is indeed relatively prominent in Sudan with a correlation co-efficient R^2^ = 34.88, and this means that the temperature has a stronger impact on meningitis than malaria, i.e., the higher the temperature, the more the prevalence of meningitis (Table 2). There is a strong positive relationship between rainfall and malaria, with a correlation coefficient R^2^ = 71.34 and 73.56 in Mangrove swamp and Freshwater respectively in the south and a moderate association in the north (R^2^ = 67.45). There is a weak positive relationship between rainfall and meningitis in all ecological zones, with the highest and lowest correlation coefficient R^2^ of 24.32 and 10.32 in Sudan and Mangrove swamp respectively. Thus this data means that the rainfall does not have a significant influence on meningitis transmission. The relationship between malaria and temperature is positively weak, with the highest and lowest correlation coefficient R^2^ = 34.88 and 21.88 in Sudan and Mangrove, respectively. In the Sahel, Sudan and Guinea, meningitis has a strong correlation with temperature R^2^ = 78.45, 73.23 and 65.01 respectively (Table 2). The temperature in the north is always higher than the south, the north-easterly originating from the Sahara desert blows diagonally over these ecological zones. Table 2, the dust has a strong correlation with meningitis: Sahel, Sudan, and Guinea indicate a correlation coefficient R^2^ = 77.33, 62.11, and 53.89, respectively.

**Table 2:** Correlation between climatic indices and the prevalence of malaria and meningitis in different ecological zones in the study area.

Ecological	Rainfall		Temperature		Dust	
	Malaria	Meningitis	Malaria	Meningitis	Malaria	Meningitis
Sahel	67.45	23.42	33.98	78.45	22.12	77.33
Sudan	59.23	24.32	34.88	73.23	11.23	62.11
Guinea	64.54	21.34	31.90	65.01	13.23	53.89
Rainforest	71.23	14.12	24.68	45.87	19.42	34.75
Freshwater	73.56	10.98	24.54	13.98	12.34	20.86
Mangrove	71.34	10.32	21.88	41.12	10.12	30.02

In comparing the impacts of climate indices on malaria and meningitis, it was found that rainfall was directly associated (Table 3) with malaria (p = 0.001) and aerosol directly associated with meningitis (p < 0.001). The results presented in Table 3 imply that rainfall has the strongest impact on malaria occurrence in the study area while aerosol has the strongest impact on meningitis. In contrast, rainfall has the least impact on meningitis. It is obvious that there is a significant linear relationship between climatic indices and malaria/meningitis. Rainfall is significantly and positively related to malaria (p < 0.01), while temperature (p < 0.01) and aerosol (p < 0.01) are significantly and positively related to meningitis (Table 3). The results further revealed that the response variable fits the criteria with the R^2^ = 0.695 (Table 3). This implies that climatic factors explain about 70% of variations in malaria/meningitis occurrence in different ecological zones. This is the percentage of variation in the response that is explained by the model, thus the response variable fits the criteria. However, comparing the impacts of climatic variables, the coefficients reveal that rainfall has the strongest impact on malaria prevalence while temperature and aerosol have the strongest impacts on meningitis incidence but low impacts on malaria incidence (Table 3). The reasons for this scenario is very clear, the malaria parasites develop only within a certain temperature range, where the minimum temperature for parasite development lies between 14.5 °C and 15 °C in the cases of *P*. vivax and between 16 °C and 19 °C for *P. falciparum*, while the proportion of parasites surviving decreases rapidly at temperatures over 32–34 °C^28,60,61^. However, aerosol has the least impact on malaria prevalence with p < 0.10, similarly temperature has strongest impact on malaria prevalence with p < 0.01 (Table 3). The results imply that the amount of aerosol couple with the intensity of temperature in a particular ecological zone influences the transmission of meningitis^21,57^.

**Table 3:** The results of multiple linear regression analysis of climatic indices impacts on prevalence of malaria and meningitis.

Variable	Unstandardized coefficient	Standardized coefficient		Sig p-value	95.0% CI	
	B	Std. error	Beta		Lower	Upper
**Intercept**						
Malaria	1.098	0.292		0.000***	7.034	9.631
Meningitis	1.142	0.301		0.000***	6.345	10.234
**Rainfall**						
Malaria	0. 640	0.082	0.601	0.001***	0.456	0.731
Meningitis	0.146	0.036	0.123	0.032**	0.127	0.262
**Temperature**						
Malaria	0.055	0.032	0.085	0.041*	0.097	0.123
Meningitis	0.465	0.063	0.321	0.003***	0.274	0.531
**Aerosol**						
Malaria	0.016	0.012	0.061	0.021**	0.021	0.103
Meningitis	0.587	0.071	0.402	0.000***	0.344	0.611
R^2^ = 0.695	Adj.R^2^ = 0.595		F-statistic = 32.142			

This table demonstrates the multivariate regression analysis results where Malaria/ Meningitis = β0 + β1 (Rainfall) + β2 (Temperature) + ε. *, **, *** indicate significant at the 0.1, 0.05, and 0.01 levels respectively. CI, Confidence Interval.

Table 4 further illustrates the probability rates of meningitis epidemic experience for each ecological zones. Sudan reveals the highest probability of occurrence of meningitis (West 0.47, East 0.59), succeeded by the Sahel savannah (West 0.41, East 0.39), the chance decreases southwards with Guinea (West 0.23, East 0.10), Rainforest (West 0.04, East 0.03), Freshwater (East 0.27), Mangrove (West 0.11, East 0.15). The probability meningitis prevalence is higher in the Sahel than in other ecological zone (Table 4). Likewise, aerosol shows many relationships with meningitis, especially in the northern ecological compared to other zones. The result implies that meningitis is likely to occur in the months with high dust, while for the months with the lowest distribution of aerosol, meningitis will be at the lowest. These data stressed the comment of Mueller, who hypothesizes that low air humidity and high aerosol load influenced nasopharyngeal colonization and the number of asymptomatic *Neisseria meningitidis*, often commonly referred to as meningococcus, carriers^24^. In general, the epidemic of meningitis depends on the prevalence of carriage in the population. Therefore, climate change via the route of transmission influenced the trend of meningitis. Earlier Studies revealed that variations in temperature and aerosols have significant impacts on the meningitis occurrences^57,62,63^. The study revealed that the cases of meningitis would increase by 6 and 9 for every increase of 1 °C in temperature^63^. The present study, accordingly, revealed that meningitis occur during the dry season with the high temperature^21,64^, and the presence of dust (aerosols) also affect the probability of meningitis occurring^57^. This is obvious as meningitis is a transmittable disease with bacteria that are readily transferred from one person to the other^65^. It is thus thought the seasonality of meningitis is related to increased invasiveness of the pathogen rather than from person to person transmission and the invasiveness of this pathogen is widely determined by climate, which includes dust, extreme dryness and host’s mucosal defences^62,66^.

**Table 4:** Observed meningitis in historical record and predicted probability of meningitis epidemic.

Ecological zones	Observed meningitis in historical record		Predicted probability of meningitis epidemic experience	
	West	East	West	East
Sahel	2	2	0.41	0.39
Sudan	2	2	0.47	0.59
Guinea	2	2	0.23	0.10
Rainforest	2	0	0.04	0.03
Freshwater	–	2	–	0.27
Mangrove	2	2	0.11	0.15

Limitations of the present study are apparent. Besides the nature of the analysis, in terms of simple correlation analysis, there is high likelihood of other confounding factors that may affect MM. The factors affecting MM epidemics or distribution cannot be limited to climate alone. There are non-climatic factors, whether directly or indirectly, that have a significant relationship with the occurrence of these epidemics. These factors that affect malaria include irrigation, agricultural practices and other land use practices^1,67^. According to Lindblade et al.^67^, the impacts of land use change on malaria transmission in the south‐western highlands of Uganda, and that increased temperatures are responsible for elevated malaria transmission risk in cultivated areas. The migration of non-immune people creates an environment for the epidemic. These areas are unplanned, and poor people live in unsanitary condition. This creates the right environment for getting malaria. Urbanization increases both malaria and meningitis, the overpopulation in urban centres which causes a rapid transmission of the diseases, aerosols are generated more in urban centres, and there is a higher probability of having water-logging areas especially in unplanned urban centres that increase the breeding of mosquitoes^68,69^. Also, Irrigation can increase the transmission season of malaria. Irrigation is created by building large numbers of dams and canals which often cause seepages from carnal and the rise in the water table, thus creating a source of still water in which malaria vectors can breed^70,71^. Dry regions require moisture to carry out agricultural practices, so pipes and drains are created to move water to the farm-sites. Leaks in the pipes and tubes rupture create artificial water bodies which serve as a breeding ground for mosquitoes, and this seems to be contradictory to meningitis occurrence, because meningitis is predominant in dry regions due to the presence of aerosols. This present study concluded that accessibility to climate information^33,72^, healthcare facilities with adequate health provision is a fundamental determinate for better health care at instance of climate change and prevalence of the outbreaks will be minimal.

## Conclusion

The seasonal prevalence of malaria and meningitis in different ecological zones of Nigeria were examined in this study. The major findings of this study is that the climatic indices have significant influence on the event and spread of MM. Because of the nature of it variability or rainfall in amount and intensity in different ecological zones, rainfall is considered to be one of the significant factors influencing variability in malaria transmission, only because it provides a breeding site for mosquitoes (malaria vector) to lay their eggs and ensure a suitable humid condition to prolong the mosquito survival. Temperature determines the ability of mosquitoes to survive as they cannot survive under extreme temperature conditions. Aerosols, which consist of dust, gases, water vapor determines meningitis prevalence to no small extent. The climatic variable that has the most substantial influence on meningitis is aerosol. But, the prevalence of malaria is higher in the south than in the north; the reason for this is that there is more precipitation in the south than in the north of Nigeria.

The influence of several climatic variables on worldwide droplets-transmitted diseases such as meningitis are discussed in multiple geographical areas such as African area^40,57,73^. Still, Wang et al^74^ have outlined as wind speed and changes in tails of the distribution are crucial for understanding the winds’ influence on dust emission. In the present study, we showed as meningitis transmission is significantly influenced by dust (aerosol) but not always by rainfall. Recently, the comparison between Italian city with low and high wind speed as coast city that are exposed to consistent winds suggesting as the air pollution and meteorological condition have influenced SARS-CoV-2 transmission and among these variables also wind speed should be considered^75^. In a not yet peer-reviewed paper, Coccia delineates that the accelerate and vast diffusion of COVID-19 in Northern Italy has a high association with air pollution and hinterland cities have average days of exceeding the limits set for PM10 equal to 80 days, while coastal cities have days of exceeding the limits set for PM10 equal to 60 days. Remarkably, the average number of COVID-19 infections was more than 2,000 individuals as of April 1st, 2020 in the hinterland cities, while the average number of these infections was about 700 in the coastal cities^75,76^. Coccia’s^75^ data further point to two key mechanisms including the air pollution-to-human transmission and the human-to-human transmission to explain his data. He suggests that to minimize future epidemic similar to COVID-19, the max number of days per year in which cities can exceed the limits set for PM10 or for ozone should be less than 50 days. Therefore, we suggest that precipitation, temperature and aerosol are significant climatic drivers that must be considered in predicting the occurrence and migration of human disease such as malaria and meningitis.
